# Adverse effects of yoga: a national cross-sectional survey

**DOI:** 10.1186/s12906-019-2612-7

**Published:** 2019-07-29

**Authors:** Holger Cramer, Daniela Quinker, Dania Schumann, Jon Wardle, Gustav Dobos, Romy Lauche

**Affiliations:** 1 Department of Internal and Integrative Medicine, Kliniken Essen-Mitte, Faculty of Medicine, University of Duisburg-Essen, Am Deimelsberg 34a, 45276 Essen, Germany; 2 Australian Research Centre in Complementary and Integrative Medicine (ARCCIM), University of Technology Sydney, Sydney, Australia

**Keywords:** Yoga, Injuries, Adverse effects, Safety, Epidemiology

## Abstract

**Background:**

While yoga is increasingly used for health purposes, its safety has been questioned. The aim of this cross-sectional survey was to analyze yoga-associated adverse effects and their correlates.

**Methods:**

A cross-sectional anonymous national online survey among German yoga practitioners (*n* = 1702; 88.9% female; 47.2 ± 10.8 years) was conducted from January to June 2016. Participants were queried regarding their yoga practice, i.e. yoga styles used, length and intensity of yoga practice, practice patterns, and whether they had experienced acute or chronic adverse effects of their yoga practice. Independent predictors of acute or chronic adverse effects were identified using multiple logistic regression analyses.

**Results:**

Ashtanga yoga (15.7%), traditional Hatha yoga (14.2%), and Sivananda yoga (22.4%) were the most commonly used yoga styles. 364 (21.4%) yoga users reported 702 acute adverse effects, occurring after a mean of 7.6 ± 8.0 years of yoga practice. The most commonly reported yoga practices that were associated with acute adverse effects were hand-, shoulder- and head stands (29.4%). Using Viniyoga was associated with a decreased risk of acute adverse effects; practicing only by self-study without supervision was associated with higher risk. One hundred seventy-three participants (10.2%) reported 239 chronic adverse effects. The risk of chronic adverse effects was higher in participants with chronic illnesses and those practicing only by self-study without supervision. Most reported adverse effects concerned the musculoskeletal system. 76.9% of acute cases, and 51.6% of chronic cases reached full recovery. On average 0.60 injuries (95% confidence interval = 0.51–0.71) per 1000 h of practice were reported, with Power yoga users reporting the highest rate (1.50 injuries per 1000 h; 95% confidence interval = 0.98–3.15).

**Conclusions:**

One in five adult yoga users reported at least one acute adverse effect in their yoga practice, and one in ten reported at least one chronic adverse effect, mainly musculoskeletal effects. Adverse effects were associated with hand-, shoulder- and head stands; and with yoga self-study without supervision. More than three quarters of of cases reached full recovery. Based on the overall injury rate per 1000 practice hours, yoga appears to be as safe or safer when compared to other exercise types.

**Electronic supplementary material:**

The online version of this article (10.1186/s12906-019-2612-7) contains supplementary material, which is available to authorized users.

## Background

Although traditionally rooted in Indian philosophy, yoga has been gaining popularity worldwide, with few signs that its popularity will decrease soon [1–4]. One country where this trend has been observed is in Germany, where there are about 6000 yoga studios [5]. The lifetime prevalence of yoga use in Germany in 2018 was 16% [6]; with the point prevalence increasing from 3% in 2014 to 5% in 2018 [4, 6]. Yoga is primarily used for health maintenance and preventive purposes, but is also increasingly being used for the treatment of specific physical and mental health conditions. Such conditions include chronic back [7–9] and neck pain [10, 11], cancer related conditions [12–14], stress [15], and depression [16]. In Germany, yoga is used for increasing physical and mental well-being by 62.9 and 56.9%, respectively [4], with spiritual reasons for practice reported by 29.4% [4]. While a large volume of research has reported benefits of yoga for health and well-being for a variety of these conditions, research on the safety profile of yoga remains relatively sparse.

Single case reports on yoga-related injuries have been published as early as 1969 [17]. However more rigorous research of the safety of yoga gained momentum only after the publication of William Broad’s book, and a related news story in the New York Times, which described a number of serious incidents in relation to yoga practice [18, 19]. While this work did not scientifically assess the entirety of yoga-related safety data available, subsequent studies and reviews have been conducted in an attempt to fill this research gap. Systematic reviews have summarized yoga safety findings from case reports [17], longitudinal studies [20], and randomized controlled trials [21]. Further cross-sectional studies have also been conducted to capture adverse reactions reported by yoga users themselves [22–24], or by data collected routinely in emergency departments [25, 26]. However, to date no data on yoga-associated adverse events in Germany were available.

While these studies provide important insights into the nature of yoga-related adverse effects, there is a paucity of specific detail on the risk profile of specific individual yoga practices. This is largely the case because most studies do not report the styles of yoga related to adverse effects, the specific exercises associated with adverse effects, the incidence rates of adverse effects, or whether the injuries were temporary (allowing affected individuals to fully recover from the adverse effects) or permanent in nature. In order to fill this important research gap, and to provide a comprehensive analysis of yoga-related adverse effects, this paper reports the findings of a cross-sectional study of adverse events conducted among German yoga practitioners.

## Methods

### Design and participants

An anonymous national online survey was conducted from January to June 2016 using the online platform SoSci Survey (https://www.soscisurvey.de). Data from this survey were used in a prior analysis [27]. An English language translation of the complete survey can be found in Additional file 1. Participants were recruited by email from national yoga teachers’ associations, organizers of yoga congresses and yoga studios. A total of 4 yoga teachers’ associations, 3 congress organizers, and 145 yoga studios were contacted and asked to send the link of the survey to their members or customers. All participants aged 18 years or older who currently practiced yoga were eligible for the survey. Ethics approval was gained from the ethics committee of the University Hospital Essen (approval number: 15–6607-BO) prior to the start of the survey. The survey included questions on sociodemographic and yoga practice characteristics, health-related variables (reported elsewhere [27]), and adverse effects. A total of 1702 participants completed the survey.

### Sociodemographic and yoga practice characteristics

The survey collected sociodemographic data such as age, gender, marital status, education, and employment status. It also collected data on the presence or absence of chronic illness, and on the number of chronic illnesses if applicable.

Participants were further queried regarding the yoga style they primarily practiced (one style could be chosen from a dropdown menu or entered as free text) and whether they used props (such as belts, blocks or blankets) as part of their yoga practice. Participants were asked how long ago they had started practicing yoga, and whether they were practicing at yoga classes, at home (repeating what they learned in class), and/or as self-study without any current or prior supervision. The frequency of yoga practice (times per week or month) and the average duration of practice were assessed for both home practice and supervised practice. Participants were also asked to indicate the proportion of their total yoga practice spent on yoga poses, breathing exercises, meditation, relaxation, philosophy (i.e. lectures on the philosophy of yoga or reading books, watching videos etc. on the philosophical background of yoga) and other yoga components. For each variable, the practice frequency was calculated as minutes per week.

### Adverse effects associated with yoga practice

Participants were queried on whether they had ever experienced an acute injury or other adverse effect during yoga practice. Participants were informed that this category of adverse effects should only include events that occurred suddenly in a specific yoga practice situation. Specifically, the participants were asked: “Have you ever experienced an acute injury or other acute complaint during yoga practice? (Note: here, adverse effects should be listed that occurred suddenly in a specific yoga practice situation).” If participants indicated that they had experienced an adverse event, they were also asked to report the number of such events. Participants were asked to name up to five specific adverse events as a free text (starting with the most severe one). For each adverse event they were asked to indicate a) during which specific yoga practice they occurred, b) whether they reached full, partial, or no recovery, c) for how long they had practiced yoga when the adverse effect occurred, and d) whether the adverse effect occurred during supervision by a yoga teacher/therapist, during home practice (repeating what they learned in class), or during self-directed practice without any current or prior supervision.

Participants were also asked to indicate whether they had ever experienced chronic adverse effects due to their yoga practice. This category of effects was defined as adverse effects that occurred or aggravated over a longer period of time and was associated with longer-term yoga practice. Specifically, the participants were asked: “Have you ever experienced other complaints associated with your yoga practice? (Note: here, adverse effects should be listed that occurred over time through repeated yoga practice or aggravated over the years).” Again, the number of such events was queried, as was the nature of up to five specific adverse events (starting with the most severe one). Participants were asked to indicate a) whether they reached full, partial, or no recovery, b) for how long they had practiced yoga when the adverse effect occurred, and c) whether the adverse effect occurred during supervision by a yoga teacher/therapist, during home practice (repeating what they learned in class), or during self-directed practice without any current or prior supervision.

### Statistical analyses

Analyses were conducted for all participants who completed the survey. Sociodemographic and yoga-related data were expressed as means, standard deviations and range or frequencies and percentages as appropriate. Bivariate associations of primary yoga style and the use of props with acute or chronic adverse effects were analyzed by Chi-squared tests. Independent predictors of adverse effects were identified using forward stepwise multiple logistic regression analyses. Adjusted odds ratios with 95% confidence intervals were computed; quartiles were calculated for longitudinal variables. Analyses were adjusted for age, gender, marital status, education, and employment. All statistical analyses were performed using IBM SPSS® software (IBM SPSS Statistics for Windows, release 22.0. Armonk, NY: IBM Corp.).

## Results

### Participants

A total of 1702 participants completed the online survey. Sociodemographic and yoga practice characteristics of participants are presented in Table 1 and Table 2, respectively.

**Table 1:** Sociodemographic characteristics of participants

Age (in years), mean ± standard deviation	47.24 ± 10.79
Gender: female, n (%)	1498 (88.9%)
Marital status: married / in a relationship, n (%)	1193 (70.1%)
Education, n (%)	
No qualification	3 (0.2%)
Secondary modern school (“Hauptschule”)	50 (2.9%)
High School (“Realschule”)	359 (21.1%)
A-Level diploma (“Abitur”)	369 (21.7%)
University degree	877 (51.5%)
Other	44 (2.6%)
Employment, n (%)	
Full time	710 (41.7%)
Part time	534 (31.4%)
House keeper	60 (3.5%)
Unemployed	15 (0.9%)
Retired	126 (7.4%)
Student	41 (2.4%)
Other	183 (10.8%)
Chronic illness	561 (33.0%)
Number of chronic illnesses (in the subsample of participants with chronic illnesses), mean ± standard deviation	1.64 ± 0.95

**Table 2:** Yoga practice characteristics

Primary yoga style (alphabetical order), n (%)	
Ashtanga Yoga	267 (15.7%)
(Traditional) Hatha Yoga	241 (14.2%)
Iyengar Yoga	143 (8.4%)
Kundalini Yoga	186 (10.9%)
Krishnamacharya Tradition / Viniyoga	161 (9.5%)
Power Yoga	71 (4.2%)
Sivananda Yoga / Yoga Vidya	381 (22.4%)
Others	252 (14.8%)
Use of props	1074 (63.1%)
Practices yoga since (in years), mean ± standard deviation	12.72 ± 9.95
Practice location, n (%)	
Yoga classes (as a student)	1250 (74.1%)
At home (repeating what learned at class)	482 (28.6%)
At home (self-study)	1026 (60.8%)
Weekly yoga practice (in minutes), mean ± standard deviation	
Total	249.79 ± 184.38
Location	
In class	84.81 ± 98.58
At home	166.26 ± 174.42
Practice components	
Yoga poses	124.51 ± 99.72
Breathing exercises	32.88 ± 35.56
Meditation	39.99 ± 53.54
Relaxation	25.81 ± 24.81
Yoga philosophy	24.98 ± 36.53

### Adverse effects associated with yoga practice

**Fig. 1:**
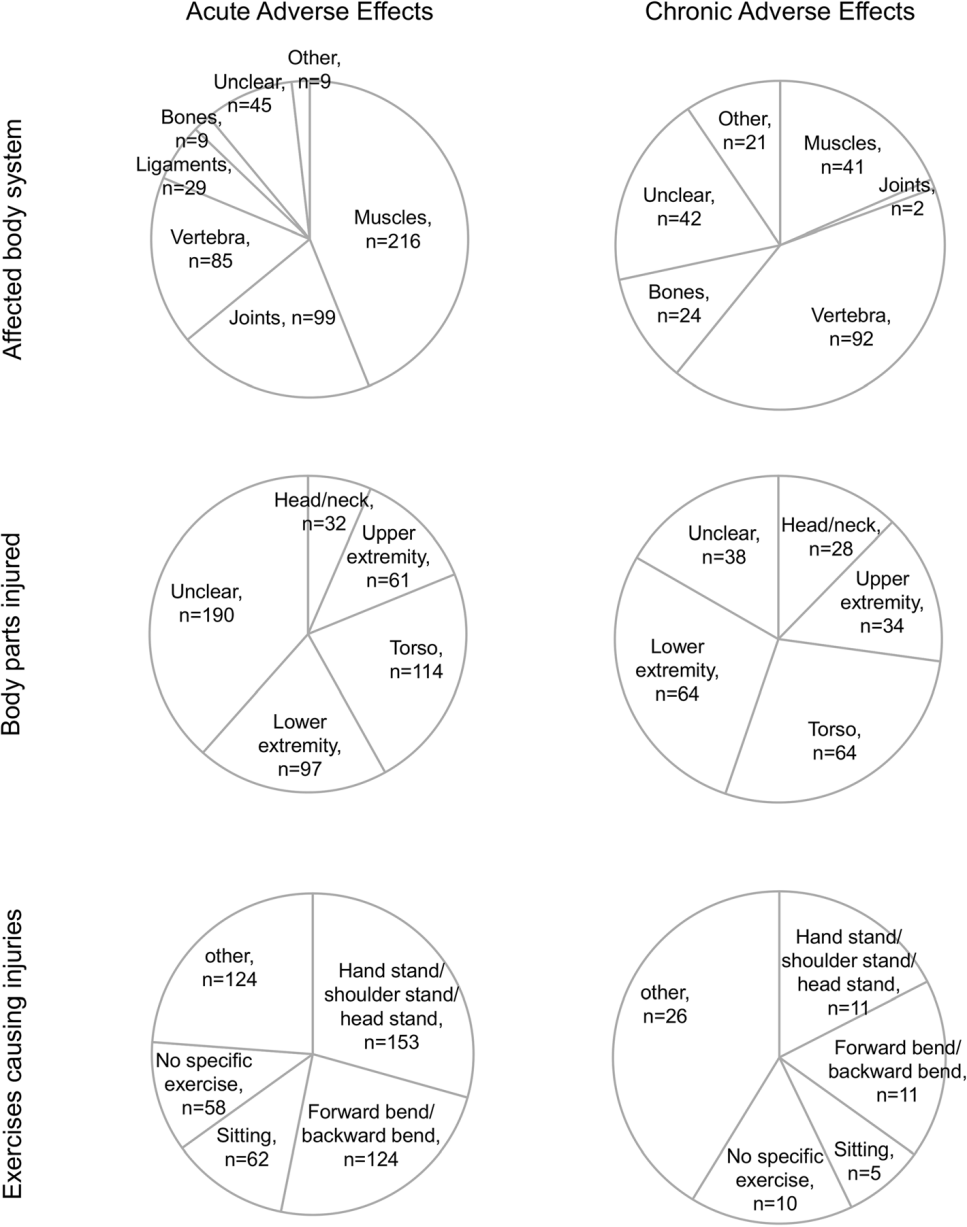
Classification of acute and chronic adverse effects regarding the affected body system, the injured body parts, and the exercises associated with the injuries

Out of 1702 participants who completed the survey, 364 (21.4%) reported a total of 702 acute adverse effects. Acute adverse effects occurred after 7.6 ± 8.0 years of yoga practice on average. Almost all reported acute adverse effects were associated with the musculoskeletal system (98.2%; Fig. 1). The most commonly reported yoga practices that were associated with acute adverse effects were hand-, shoulder- and head stands (29.4%), forward and backward bends (23.8%), and sitting positions (11.9%) (Fig. 1).

**Fig. 2:**
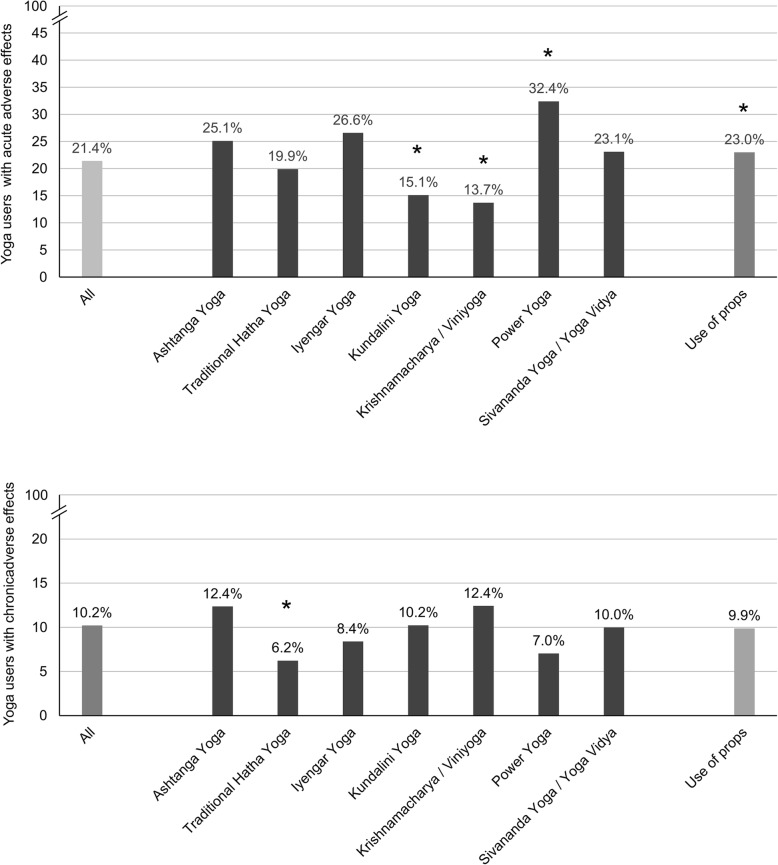
Rates of acute and chronic adverse effects by yoga style and props use (*n* = 1702). Asterisks indicate significantly higher or lower adverse effects compared to all other participants

Compared to other yoga styles, acute adverse effects were more common in participants using power yoga as their primary yoga style (*p* = 0.026), and less common in participants using Kundalini yoga (*p* = 0.026) or Viniyoga as their primary yoga style (*p* = 0.011, Fig. 2). Out of 1074 participants using props as part of their yoga practice, 247 (23.0%) reporting acute adverse effects associated with their yoga practice compared to 117 (18.6%) of the 628 participant not using props (*p* = 0.037, Fig. 2). Of acute adverse effects 55.2% occurred during supervision by a yoga teacher/therapist, 22.2% during home practice (repeating what they learned in class), and 22.6% during self-directed practice without any current or prior supervision. 76.9% of cases reached full recovery, 19.5% reached partial recovery, and 3.7% reached no recovery.

In logistic regression analysis, the use of Viniyoga as primary yoga style was independently associated with a decreased risk of acute adverse effects (Table 3). Practicing only by self-study without prior or current supervision by a yoga teacher/therapist was independently associated with a higher risk of acute adverse effects, as was a higher practice of yoga philosophy (Table 3). Sociodemographic variables were not independently associated with risk of acute adverse effects.

**Table 3:** Predictors associated independently with acute and chronic adverse effects. Only those categories of predictors are shown that were significantly associated with acute or chronic adverse effects

Dependent variable	Predictor variable	Adjusted odds ratio (95% confidence interval)
Acute adverse effects	Chronic illness	1.78 (1.37–2.31)
	Primary yoga style	
	Asthanga Yoga	1.43 (0.92–2.25)
	(Traditional) Hatha Yoga	1.09 (0.68–1.75)
	Iyengar Yoga	1.49 (0.88–2.51)
	Kundalini Yoga	0.60 (0.35–1.04)
	Krishnamacharya Tradition / Viniyoga	0.54 (0.30–0.99)
	Power Yoga	1.88 (0.99–3.56)
	Sivananda Yoga / Yoga Vidya	1.06 (0.69–1.62)
	Other	Reference
	Home practice (self-study)	1.75 (1.31–2.33)
	Weekly practice frequency: meditation	
	First quartile	Reference
	Second quartile	0.92 (0.64–1.32)
	Third quartile	0.60 (0.40–0.92)
	Fourth quartile	1.01 (0.66–1.54)
	Weekly practice frequency: philosophy	
	First quartile	Reference
	Second quartile	1.27 (0.87–1.86)
	Third quartile	1.31 (0.87–1.97)
	Fourth quartile	2.00 (1.32–3.03)
Chronic adverse effects	Chronic illness	1.44 (1.02–2.02)
	Home practice (self-study)	1.72 (1.20–2.47)

A total of 173 participants (10.2%) reported 239 chronic adverse effects. The most commonly reported types of chronic adverse effects concerned the musculoskeletal system. These included osteoarthritis, chronic back, neck or shoulder pain, tendon shortening or sciatica (90.5%; Fig. 1). Other chronic adverse effects included chronic headache, sleep problems, or depressive symptoms. Compared to other yoga styles, chronic adverse effects were less common in participants using traditional Hatha yoga as their primary yoga style (*p* = 0.029, Fig. 2). Adverse effects occurred after a mean of 7.3 ± 7.7 years of yoga practice. Of all chronic adverse effects, 52.0% were associated with supervised yoga practice, 28.0% with home practice (repeating what they learned in class), and 20.0% with self-directed practice without any current or prior supervision. 51.6% of chronic adverse event cases reached full recovery, 33.3% reached partial recovery, and 15.1% reached no recovery.

Logistic regression suggested the risk of chronic adverse effects was higher in participants with chronic illnesses and/or those practicing only by self-study without prior or current supervision by a yoga teacher/therapists (Table 3). Sociodemographic variables were not independently associated with risk of chronic adverse effects.

Analyses revealed that on average 0.60 acute injuries per 1000 h of practice were reported by the study participants (95% confidence interval [CI] = 0.51–0.71), with Power yoga users reporting the highest rate (1.50/1000 h; 95% CI = 0.98–3.15), while other types of yoga were found to have a comparably low rate of injuries: Sivananda yoga (0.63/1000 h; 95% CI = 0.45–1.03), Vinyasa yoga (0.61/1000 h,; 95%CI = 0.47–0.90); Iyengar yoga (0.52/1000 h; 95%CI = 0.37–0.88, Kundalini yoga (0.59/1000 h; 95%CI = 0.40–1.13); and ‘other’ yoga styles (0.48/1000 h; 95%CI = 0.31–1.01).

While the majority of acute adverse effects were considered minor, and included events such as strains and sprains, 16 (2.3%) acute adverse effects had to be classified as serious, including one case of cerebral hemorrhage, and multiple cases of fractures, spinal injuries, and nerve injuries. All chronic adverse events were classified as minor.

## Discussion

### Adverse effects rate

This is the first study reporting adverse effects of yoga in German yoga users. Previous studies have been conducted internationally, and have reported injury prevalence ranging from 2.4% (Australia) [24] to 62% (Finland, 110 participants surveyed) [23]. The differences in reported injury prevalence rates are significant, and are likely to be due to the survey format and the time frame in which participants experienced adverse effects.

Analysis of the injuries from yoga users in our study has also revealed that on average 0.60 injuries were reported every 1000 h of yoga practice, with large differences between the yoga styles. Power yoga, a physically demanding yoga style using flowing sequences of yoga postures, was found to be the most associated with adverse effects and was associated with 1.50 injuries per 1000 h of practice in our study. Similar rates of injuries have been reported previously [23]. A common factor with the yoga types associated with the most adverse effects in our study was that they emphasized postures over other aspects such as meditation or breathing exercises (or at least promoted more vigorous physical postures). Focusing on physical postures at the expense of other fundamental components of whole-practice yoga has been criticized as reductionist and incompatible with traditional practices [28], and emphasizing the importance of non-physical aspects of yoga to align with traditional practice may be one way to reduce the risk of adverse effects.

Compared to other types of sports and exercises, however, the overall injury rate of yoga per 1000 practice hours appears to be relatively low. Previous studies reported incidence rates from 2.5 injuries per 1000 h for general cardiovascular fitness activities [29] or running [30], to 3.7 injuries per 1000 h for soccer [31], to 5.0 injuries per 1000 h for tennis [29] and 8.0 injuries per 1000 h for skiing [29]. Strongman or strength athletics were reported to result in 4.5–6.1 injuries per 1000 h, and highland games in 7.5 injuries per 1000 h of practice [32]. These figures suggest that yoga appears to be as safe or safer when compared to other exercise types.

### Types of adverse effects

The present study found that the vast majority of adverse effects from yoga affected the musculoskeletal system. These findings are mostly in line with findings from previous studies, which mainly reporting muscle or joint pain or strains [22, 24]. The present study however also found several serious adverse effects, ranging from joint injuries to bone fractures and disc prolapse, which may not be amenable to full recovery, potentially affecting longer-term health and well-being.

This study was the first to assess whether participants had recovered from their reported injuries. Findings from our study suggest that nearly one in four participants with acute injuries resulting from their yoga practice - and more than half of those with chronic adverse effects from their yoga practice – reported that they did not fully recover from their injuries. A previous study found that the number of yoga injuries requiring medical attention has been rising in the past decades [25], and emergency departments reported injuries to muscles and soft tissues, fractures, contusions and dislocations. While the number of severe injuries associated with yoga is relatively small (4.6% requiring medical treatment) [24], these findings suggest further attention on yoga-associated adverse events is warranted, and further studies are needed to identify the circumstances leading to those severe injuries, as well as to examine and identify effective ways in which such injuries can be avoided.

While the vast majority of adverse effects reported in our study were musculoskeletal, some participants in this study also reported adverse effects affecting other areas, including one case of a cerebral hemorrhage. Other adverse effects that have been reported in case reports and cross-sectional studies included injuries to the eyes, for example in participants with preexisting glaucoma [17, 20]. It is possible that adverse effects other than musculoskeletal injuries may not have been recognized as such by participants, and have been underreported due to the unclear association to yoga practice (for example due to the delayed manifestation of symptoms). It may be prudent to encourage physicians to include yoga in the list of physical activities undertaken when collecting patient histories, to ensure that any relevant yoga-related outcomes can be more accurately captured.

### Predictors of adverse effects

One of the identified predictors for injuries among participants of our study was the specific yoga style practiced by participants, with vigorous forms of yoga being associated with higher risk of injuries. Vigorous yoga forms often combine postures into a series of movements, which could result in higher load on muscles, ligaments and joints compared to slow and more meditative yoga styles [20]. These more vigorous styles of yoga may also include higher frequencies of specific yoga postures found to be the cause of a large number of injuries, including hand stands, head stands or shoulder stands, forward and backward bends. This finding is supported by another study, [24] which reported the same exercises as the most common triggers of yoga adverse effects. Such exercises probably place large weights on body parts, for example the wrists for reversed positions or the knees in positions that require prolonged kneeling or flexion. As a result participants without sufficient preparation or training may experience pain or even injuries to the affected joints. Some studies even suggest that yoga practice may lead to meniscal damage [33], which is a risk factor for osteoarthritis and related disability [34]. However, a cross-sectional study conducted in Australia did not find higher rates of knee or other joint problems in yoga users as compared to yoga non-users [35], and such disparate findings indicate further research is warranted. Given that the joint load has been estimated based on correct execution of the exercise, with the assumption of a normal weight practitioner with no preexisting physical impairment, further studies are also needed to examine the joint load in everyday practitioners.

Another factor associated with increased risk of adverse effects among participants in our study is the presence of preexisting medical conditions or illnesses, including predispositions for certain injuries. This result confirms findings from previous studies [22, 24], showing that poor physical health and chronic disease significantly increase the risk of injuries during yoga.

Another important finding for our study is that yoga self-study without prior or current supervision is more likely to lead to adverse effects than supervised yoga practice. Self-study is an important part of yoga practice, and is often promoted in clinical trials to increase the total practice frequency [7–9, 11–13, 36]. However, there is also a plethora of self-practice DVDs and videos, or online courses available for those who want to learn and practice yoga by themselves. Physically demanding yoga postures and motion sequences may require surveillance by experienced instructors to ensure correct execution. Self-practicing individuals may execute postures incorrectly, or push themselves too hard to follow the instructors, thus increasing the risk for injuries. These results suggest that some form of regular or formal supervisory guidance may be beneficial for reducing adverse events associated with yoga practice. An interesting finding related to this point form our study is that participants practiced in class for only 84.9 min per week but for 166.3 min at home. This is most likely the case because not all participants actually attended any classes, with some practicing only at home.

Props, such as blocks and belts have been heavily discussed in the literature as being either beneficial [37] or hazardous [19]. Props were introduced into modern yoga practice to allow practitioners to access the benefits of yoga postures regardless of their physical condition or experience [37]. In our bivariate analysis the use of props slightly increased the frequency of acute adverse effects. However, in the logistic regression no associations between the use of props and injuries were found. As such the use of props cannot be considered hazardous in general, however precautions should be applied when practicing with props, such as ensuring correct handling of props (including securing the props when they are not used), and not applying props to push and exceed bodily limitations, to reduce potential yoga-associated adverse events.

It could also be expected that injury rates differ by motivations for yoga practice, particularly if those who are motivated for fitness reasons are drawn to more vigorous forms of yoga. In this survey, more than 60% of participants were currently practicing yoga for general prevention or stress management and only 1.5% for fitness reasons; however, motivation for yoga practice was not associated with injury rates (data not shown). Comparable to other forms of physical activity, the risk of injuries did not differ between genders [32].

### Limitations

While the sample in our study comprised predominantly female participants with higher educational degrees, and is thus not representative of the general population, it may be representative of yoga users given that women are more likely to practice yoga in general. For example the reported ratio of female to male yoga users in the US was 3:1 [1], and almost 9:1 in Germany [4], indicating that the proportion of women in this sample is a relatively close reflection of their proportion in yoga practitioners in Germany. A further potential limitation of the survey is that it is based on self-reported data with no limitation regarding the time point of injury, and as such not all injuries may have been recollected and reported. On the other hand, not limiting the time period allowed the calculation the lifetime prevalence of yoga-associated adverse effects. Assessing a period prevalence instead might have resulted in a too low number of events to be able to calculate relationships. Additionally, since a snowball system was used for recruitment, the response rate cannot be determined. The increased risk of acute adverse effects with increasing yoga philosophy study is counterintuitive and difficult to interpret.

### Practical implications

The findings of this study have a number of practical implications for a safe yoga practice. Firstly, individuals with preexisting chronic conditions need to be cautious, and should preference yoga classes led by certified and experienced practitioners. Specialized yoga therapy classes might be preferable over standard yoga classes. It might also be wise for patients with preexisting chronic conditions to further consult with their general practitioner or specialist before taking up yoga practice. It is also recommended that individuals with specific conditions avoid specific positions (e.g. those with hypertension or glaucoma should avoid inversion poses, individuals with joint problems should avoid extreme twists etc.). Our findings also highlight the importance of qualified yoga instructors, who appear to reduce the risk of adverse effects in yoga users. Beginners should learn and practice yoga under supervision, and only self-study when they feel confident and are capable to execute postures correctly and safely. Finally, props may be used to improve safety, for example to support individuals in exercises requiring balance, however they should be used sparingly and not solely as a means to overcome physical limitations.

## Conclusions

One in five adult yoga users experienced at least one acute adverse effect due to their yoga practice. One in ten reported at least one chronic adverse effect, mainly musculoskeletal effects. Adverse effects seem to be mainly associated with hand-, shoulder- and head stands; and with yoga self-study without supervision. Based on the overall injury rate per 1000 practice hours, yoga appears to be as safe or safer when compared to other exercise types.

## Additional file

Additional file 1: English translation of the complete survey. (DOCX 30 kb)

## Data Availability

The data are available from Dr. Holger Cramer upon request.
