# Supplementary material for: Adverse effects of yoga: a national cross-sectional survey
Source: BMC Complement Altern Med. 2019 Jul 29;19:190. doi: 10.1186/s12906-019-2612-7 (PMC6664709; doi:10.1186/s12906-019-2612-7)
Supplement: Supplementary file 1 — English translation of the complete survey. (DOCX 30 kb) [file 12906_2019_2612_MOESM1_ESM.docx]

**Survey on yoga practice**

Dear Participant,

We are delighted that you are taking part in our study on yoga practice in Germany and would like to thank you very much in advance.

This survey is about whether yoga has an influence on physical and mental health, about why you started yoga and why you continue to practice it. You will also be asked questions on whether you have been injured during yoga practice and if so, how often. The aim of this study is to examine the relationship between yoga and your health and the possible risks of yoga.

Your participation in the survey is voluntary. You can cancel the survey at any time without giving any reason.

The survey is anonymous. No personal data, such as name, address, e-mail address or telephone numbers, will be requested. The records of your collected data will be kept strictly confidential at all times. The persons entrusted with data verification are obliged to maintain strict confidentiality and to comply with data protection. With your consent, you agree that data about your health collected in the context of this survey may be recorded and used anonymously without mentioning your name.

We would like to thank you very much for your cooperation!

**Consent form**

1. Do you agree to the anonymous use of your responses in this study?

Note: No names, addresses or other personal data will be collected.^^[[1]](#footnote-1)^^

- Yes
- No

*If yes: Continue the questionnaire*^^[[2]](#footnote-2)^^

*If no: Cancel the questionnaire*

**Introductory questions**

1. Are you currently practicing yoga?

- Yes *(complete questionnaire)*
- No, never *(cancel the questionnaire)*
- No, no more *(cancel the questionnaire)*

1. Are you a trained yoga teacher?

- Yes
- No

**Personal data**

1. Your sex

- Female
- Male

1. Your age ______ years
2. Your height ______cm
3. Your body weight _____kg
4. Your marital status

- Single
- Living with a partner
- Married
- Divorced
- Separated
- Widowed

1. Your nationality

- German
- Other: ________

1. What is your highest educational attainment?

- Not yet completed (student)
- Left school without graduation
- ‘Hauptschule’ degree / ‘Volksschule’ degree
- ‘Realschule’ degree /,’mittlere Reife’
- ‘Abitur’ / ‘Fachabitur’ (without subsequent studies)
- ‘Abitur’ / ‘Fachabitur’ with uncompleted studies
- University / University of Applied Science degree
- Other: _______

1. Are you currently employed?

- Full time
- Part-time
- Occasionally / irregular
- Unemployed
- Retired at regular retirement age
- Retired early for health reasons
- House keeper
- Student / trainee
- Other: ________________

1. Which denomination do you belong to?

- Christianity
- Judaism
- Islam
- Buddhism
- Hinduism
- Atheism / no religion
- Other: _______

**General questions about your yoga practice**

1. For how long have you been practicing yoga?

For _________ years and ____ months

1. Where do you practice yoga? (Multiple answers possible)

- Yoga studio under the guidance of a yoga teacher
- At home according to the instructions of a yoga trainer (repeating the exercises learned at the class)
- Self-study at home
- Other: ____________

1. What is your primary yoga style?

- Power Yoga
- Ashtanga
- Iyengar Yoga
- Bikram Yoga
- Sivanada Yoga
- Kundalini Yoga
- Other: _____
- Don't know

1. Do you practice other yoga styles? *(Multiple answers possible)*

- Power Yoga
- Ashtanga
- Iyengar Yoga
- Bikram Yoga
- Sivanada Yoga
- Kundalini Yoga
- No further yoga styles
- Other: _____
- Don't know

1. How many people on average attend your yoga classes? _____
2. Do you use props (such as belts, blocks or blankets) as part your yoga practice?

- Yes
- No

1. How often do you practice yoga?

- Once a week or more
- Less than once a week

1. *If once a week or more:* How often / how long do you practice on average with a yoga teacher and / or at home per week?

- With a yoga teacher: ___ times per week, for ____ minute each
- At home: ___ times per week, for ____ minute each

1. *If once a week or more:* In relation to your entire yoga practice, how is the yoga exercise time distributed among the various components of yoga? Please indicate the percentages so that you end up with 100%.

- Yoga postures (Asanas): __________%
- Breathing exercises (Pranayama): __________%
- Mediation: __________%
- Relaxation: __________%
- Theoretical / philosophical foundations of yoga (e.g. reading books, discussions in yoga classes): __________%

1. *If less than once a week:* How often / how long do you practice on average with a yoga teacher and / or at home per week?

- With a yoga teacher: ___ times per month, for ____ minute each
- At home: ___ times per month, for ____ minute each

1. *If less than once a week:* In relation to your entire yoga practice, how is the yoga exercise time distributed among the various components of yoga? Please indicate the percentages so that you end up with 100%.

- Yoga postures (Asanas): __________%
- Breathing exercises (Pranayama): __________%
- Mediation: __________%
- Relaxation: __________%
- Theoretical / philosophical foundations of yoga (e.g. reading books, discussions in yoga classes): __________%

1. Do you practice any component of yoga other than those mentioned above?

- Yes: ____________
- No

1. If so, how often do you practice this yoga component?

___ times per month, for ____ minutes each

**Questions about your motivation for yoga practice**

1. What was your primary motivation to start your yoga practice?

- Prevention / health promotion
- Treating a health issue
- Spirituality,
- Physician’s or therapist’s advice
- Relaxation / stress reduction
- Get fit / get into shape
- Current popularity of yoga
- Looking for a hobby
- Socializing with other practitioners

Monetary incentive from your health insurance

- Advice from friends or family
- Other reasons: _______

1. What were other reasons to start your yoga practice? (*Multiple answers possible)*

- Prevention / health promotion
- Treating a health issue
- Spirituality,
- Physician’s or therapist’s advice
- Relaxation / stress reduction
- Get fit / get into shape
- Current popularity of yoga
- Looking for a hobby
- Socializing with other practitioners
- Monetary incentive from your health insurance
- Advice from friends or family
- Other reasons: _______
- No further reasons

1. *If treating a health issue was given as a reason:* You have stated health problems as a reason to start yoga practice.
2. What were the health problems due to which you started yoga practice? ________________
3. Did your health problems change due to your yoga practice?

- Much better
- Slightly better
- No change
- Slightly worse
- Much worse

1. What is your primary motivation to continue with your yoga practice?

- Prevention / health promotion
- Treating a health issue
- Spirituality,
- Physician’s or therapist’s advice
- Relaxation / stress reduction
- Get fit / get into shape
- Current popularity of yoga
- Looking for a hobby
- Socializing with other practitioners
- Monetary incentive from your health insurance
- Advice from friends or family
- Other reasons: _______

1. What are other reasons to continue your yoga practice? (*Multiple answers possible)*

- Prevention / health promotion
- Treating a health issue
- Spirituality,
- Physician’s or therapist’s advice
- Relaxation / stress reduction
- Get fit / get into shape
- Current popularity of yoga
- Looking for a hobby
- Socializing with other practitioners
- Monetary incentive from your health insurance
- Advice from friends or family
- Other reasons: _______
- No further reasons

**Questionas about yoga and health**

1. In general, would you say your health is:

- Excellent
- Very good
- Good
- Fair
- Poor

1. Compared to before you started your yoga practice, how would you rate your health in general now?

- Much better than before starting yoga practice
- Somewhat better than before starting yoga practice
- About the same
- Somewhat worse than before starting yoga practice
- Much worse than before starting yoga practice

1. Do you suffer from one or more chronic diseases? (e.g. high blood pressure, back pain, glaucoma)

- Yes
- No

1. *If yes:* Which chronic diseases do you suffer from? Please start with the most serious one:

___________

___________

___________

*Here, 2 standardized and validated questionnaires, the abbreviated World Health Organization Quality of Life Instrument (WHOQOL-BREF)^^[[3]](#footnote-3)^^ and the Freiburg Mindfulness Inventory (FMI)^^[[4]](#footnote-4)^^, are used as part of the survey.*

**Yoga & Injuries**

1. Have you ever experienced an acute injury or other acute complaint during yoga practice? (Note: here, adverse effects should be listed that occurred suddenly in a specific yoga practice situation)

- Yes
- No

1. *If yes*: How many of such injuries did you experience? ______________
2. *If yes:* Which injuries did you experience? Please start with the most serious injury! Please use a new line for each injury. These open automatically by clicking on the ‘Enter key’.

______________

______________

______________

______________

______________

*(max. 5 lines)*

1. *If yes:* Think of your (most serious) injury and describe it:
2. What injury did you experience? ________________
3. During which specific yoga practice did the injury occur? ____________
4. Did you reach recovery from your injury?

- Yes, full recovery
- Partial recovery, some symptoms remained
- No recovery

1. For how long had you practiced yoga when the injury occurred? ___ years ____ months
2. Did the injury occur during training with a yoga teacher or during self-study?

- Yoga studio under the guidance of a yoga teacher
- At home during practice according to the instructions of a yoga trainer (repeating the exercises learned at the class)
- During self-directed practice at home without prior supervision

*Point 4 is repeated according to the number given in point 2 (max. five times). If the participant had experienced two or more injuries, the initial question is: Think of your most serious injury and describe it.*

1. Have you ever experienced other complaints associated with your yoga practice? (Note: here, adverse effects should be listed that occurred over time through repeated yoga practice or aggravated over the years)

- Yes
- No

1. *If yes*: How many of such complaints did you experience? ______________
2. *If yes:* Which complaints did you experience? Please start with the most serious complaint! Please use a new line for each complaint. These open automatically by clicking on the ‘Enter key’.

______________

______________

______________

______________

______________

*(max. 5 lines)*

1. *If yes:* Think of your (most serious) complaint and describe it:
2. What complaint did you experience? ________________
3. During which specific yoga practice did the complaint occur? ____________
4. Did you reach recovery from your complaint?

- Yes, full recovery
- Partial recovery, some symptoms remained
- No recovery

1. For how long had you practiced yoga when the complaint occurred? ___ years ____ months
2. Did the complaint occur during training with a yoga teacher or during self-study?

- Yoga studio under the guidance of a yoga teacher
- At home during practice according to the instructions of a yoga trainer (repeating the exercises learned at the class)
- During self-directed practice at home without prior supervision

**Yoga and Lifestyle**

1. How many portions of fruit or vegetables do you eat a day? ________ *(Dropdown Menu: 0-10)*
2. How often do you do exercise in a week? __________ times (Note: please do not include yoga practice in this count)
3. For how long on average do you exercise? ____________ minutes
4. Do you drink alcohol occasionally or regularly?

- Never
- Occasionally
- Regularly

1. Do you smoke?

- Yes
- No

1. Your nutritional behavior:
2. Do you eat meat, sausages or meat products?

- Yes
- No

1. Do you eat fish?

- Yes
- No

1. Do you eat eggs and/or dairy products?

- Yes
- No

1. How would you rate your sleep quality if 10 is very good and 1 very bad?

- 1
- 2
- 3
- 4
- 5
- 6
- 7
- 8
- 9
- 10

1. To what extent do you suffer from exhaustion and fatigue in everyday life?

Note: 1 = I do not suffer from exhaustion and fatigue

10 = I suffer from total exhaustion and fatigue

- 1
- 2
- 3
- 4
- 5
- 6
- 7
- 8
- 9
- 10

***Technical questions:***

1. Did anyone help you fill out this questionnaire?

- Yes
- No

1. How long did it take to complete the questionnaire? ________________ minutes
2. Do you have any comments on this questionnaire? _______________

1. Technical note; If not otherwise denoted, multiple answers are not possible. [↑](#footnote-ref-1)
2. Technical note: Italic text describes the survey structure; it is not shown to survey participants. [↑](#footnote-ref-2)
3. The WHOQOL Group: **Development of the World Health Organization WHOQOL-BREF quality of life assessment.** *Psychol Med* 1998, **28**(3):551-558. [↑](#footnote-ref-3)
4. Walach H, Buchheld N, Buttenmüller V, Kleinknecht N, Schmidt S: **Measuring mindfulness - the Freiburg Mindfulness Inventory (FMI).** *Personality and Individual Differences* 2006, **40**(8):1543-1555. [↑](#footnote-ref-4)
